# Concomitant injuries may not reduce the likelihood of achieving symmetrical muscle function one year after anterior cruciate ligament reconstruction: a prospective observational study based on 263 patients

**DOI:** 10.1007/s00167-018-4845-2

**Published:** 2018-02-05

**Authors:** Eric Hamrin Senorski, Eleonor Svantesson, Susanne Beischer, Christoffer Thomeé, Alberto Grassi, Ferid Krupic, Roland Thomeé, Jón Karlsson, Kristian Samuelsson

**Affiliations:** 1 Department of Health and Rehabilitation, Institute of Neuroscience and Physiology, The Sahlgrenska Academy, University of Gothenburg, Gothenburg, Sweden; 2 Sportrehab, Sport Medicine Clinic, Gothenburg, Sweden; 3 Department of Orthopaedics, Institute of Clinical Sciences, The Sahlgrenska Academy, University of Gothenburg, Gothenburg, Sweden; 4 Clinica Ortopedica e Traumatologica II-Lab. di Biomeccanica, ed Innovazione Tecnologica, Istituto Ortopedico Rizzoli, Bologna, Italy; 5 Department of Orthopaedics, Sahlgrenska University Hospital, Mölndal, Sweden

**Keywords:** Anterior cruciate ligament, ACL, Reconstruction, Graft, Limb symmetry index, LSI, Rehabilitation, Register, Muscle function, Sports

## Abstract

**Purpose:**

A better understanding of patient characteristics and the way common concomitant injuries affect the recovery of muscle function after surgery should help providers to treat patients with anterior cruciate ligament (ACL) injuries. The aim of this study was to determine whether patient characteristics, concomitant injuries and graft choice at ACL reconstruction were associated with symmetrical knee muscle function at one year. The hypothesis was that the presence of concomitant injuries would negatively influence the opportunity to achieve symmetrical knee function at the one-year follow-up.

**Methods:**

Data was extracted from the Swedish National Knee Ligament Register and a rehabilitation outcome register between August 2012 and December 2016. The patients had been evaluated with a battery of tests comprising knee extension and flexion strength, vertical jump, hop for distance and the side-hop test one year after ACL reconstruction. Univariable and multivariable logistic regression analyses were performed with achieving a limb symmetry index (LSI) of ≥ 90% in all tests of muscle function as primary outcome.

**Results:**

A total of 263 patients with a mean age of 26.7 ± 10.3 years were included in the study (47% females). No patient demographic or intra-operative predictors were found to be significant when attempting to predict the achievement of a symmetrical muscle function. Lateral meniscus injury and a patellar tendon autograft reduced the odds of achieving an LSI of ≥ 90% in knee extension strength, OR = 0.49 [(95% CI 0.25–0.97), *p* = 0.039] and OR = 0.30 [(95% CI 0.14–0.67), *p* = 0.0033] respectively. In addition, reduced odds of recovering knee extension strength were found in older patients, OR = 0.76 [(95% CI 0.60–0.98), *p* = 0.034]. A higher pre-injury level of physical activity increased the odds of recovering knee flexion strength, OR = 1.14 [(95% CI 1.01–1.29), *p* = 0.037].

**Conclusion:**

Intra-operatively identified concomitant injuries or graft choice did not affect the likelihood of recovering symmetrical performance in five different tests of muscle function one year after ACL reconstruction. However, fewer than one in four patients achieved an LSI of ≥ 90% in all tests.

**Level of evidence:**

Prospective observational study: Level 2.

**Electronic supplementary material:**

The online version of this article (10.1007/s00167-018-4845-2) contains supplementary material, which is available to authorized users.

## Introduction

Sustaining an injury to the anterior cruciate ligament (ACL) is something that is dreaded, especially among active athletes. However, it is well recognised that, in most cases, the current treatment for ACL tears successfully returns patients to their pre-injury level [19, 26, 37]. This in turn motivates most patients to strive for their desired comeback and work through a long period of rehabilitation. While the patient is frequently eager to return to sport (RTS), physicians and physiotherapists have important roles in guiding the patient in his or her decisions and determine readiness based on several important aspects [6]. However, RTS criteria are a complex area after ACL reconstruction and there is still no consensus on how best to determine when a patient is ready for an RTS [3, 7, 34]. One contributing explanation is that the isolated ACL injury is uncommon and the injury is instead frequently accompanied by injuries to other tissues, including articular cartilage, bone, collateral ligaments and menisci [29].

Tests of muscle function are often part of the evaluation prior to RTS and the achieved level of muscle function has been chosen as an important aspect to consider [12, 36]. When evaluating muscle function, the limb symmetry index (LSI) is the most established method. It is defined by comparing the result for the injured leg with that of the uninjured leg. It has been suggested that achieving a sufficient LSI will minimise the risk of sustaining a new injury and prevent the overuse of the other leg when returning to strenuous activity [25, 34]. However, there is limited previous research on understanding the effect of concomitant knee injuries and the ability to recover symmetrical knee function in patients after an ACL reconstruction.

A better understanding of patient characteristics and the way concomitant injuries affect the recovery of muscle function during the first year after surgery should help providers to treat patients with ACL injuries. The purpose of this study was, therefore, to determine whether patient characteristics, intra-operatively identified concomitant injuries and graft choice at primary ACL reconstruction were associated with the recovery of muscle function one year after reconstruction. Based on clinical experience, the hypothesis was that the presence of concomitant injuries would negatively influence the opportunity to achieve symmetrical knee function at the one-year follow-up.

## Materials and methods

This cohort study was based on prospectively collected data from two registers; one rehabilitation specific (Project ACL) and one surgeon specific (the Swedish National Knee Ligament Register, SNKLR). Project ACL [14] uses a web-based database for regular assessment with patient-reported outcomes and tests of muscle function for patients with an ACL injury. Assessments are performed after a predefined follow-up schedule after the index ACL injury or reconstructive surgery. The battery of tests for muscle function is conducted according to a standardised protocol and comprises an evaluation of knee strength and hop performance based on previous publications [13, 23]. At the start of the project from September 2014, isometric strength tests were performed using the David F200 and F300 DMS-EVE (David Health Solutions Ltd, 2013, Finland) and these results contribute to approximately 35% of the total strength data. The isometric test evaluated peak torque in knee extension at 60° of knee flexion and knee flexion at 30° of flexion. From December 2015, strength measurements were performed with a concentric isokinetic test of knee extension and knee flexion at 90 degrees per second using a Biodex System 4 (Biodex Medical Systems, Shirley, New York, USA) [36]. Both the isokinetic and isometric testing of knee extension and knee flexion strength has repeatedly been reported with excellent reliability, interclass correlation coefficient 0.95–0.99 and 0.93–0.99, respectively [2, 8, 31, 32]. Hop tests include a one-legged hop for distance, vertical jump (Muscle lab, Ergotest Technology, Oslo, Norway) and side-hop test. For the vertical hop and the hop for distance, the patients performed three to five practice trials, followed by three maximum trials. One attempt is allowed in the side-hop test and it was performed by the patients jumping as many times as possible over two lines 40 cm apart for 30 s. Three minutes of rest were given between legs for the side-hop test [23]. All the hop tests were performed with the patients holding their hands behind their back and the best of three attempts was recorded.

The SNKLR is a nationwide database that utilises a web-based protocol for the collection of data [1, 17]. The protocol consists of two parts; one surgeon-reported section and one patient-reported section. The operating surgeon enters information about the activity performed at the time of injury, the time from injury to reconstruction, graft selection and surgical fixation techniques. All surgical procedures performed on the injured knee, including meniscal surgery and treatment for chondral lesions, are reported. Revisions and repeated surgery for other reasons are registered as separate entries in the register [9].

### Patients

Patients in Project ACL with results from the one-year follow-up after reconstruction were eligible for inclusion. For these patients, additional intra-operative and surgical information was extracted from the SNKLR, including data on concomitant injuries and graft choice. Only patients who underwent primary unilateral ACL reconstruction and had undergone no previous knee surgery were included in the study. Patients were excluded if they had an early post-operative infection. All patients underwent an individualised criteria-based rehabilitation at their respective physiotherapy clinic. The patients received written information about the study and informed consent was obtained from included patients. Ethical approval was obtained from the Regional Ethical Review Board in Gothenburg (registration number 265-13, T023-17).

### Outcome

The LSI was used to analyse the results of the tests of muscle function and was calculated as:

$${\text{Limb}}~{\text{symmetry}}~{\text{index}}~\left( \% \right)=~\frac{{{\text{Result}}~{\text{for}}~{\text{injured}}~{\text{leg}}}}{{{\text{Result}}~{\text{for}}~{\text{uninjured}}~{\text{leg}}}} \times 100$$

Patients who had results from all five tests of muscle function at the 1-year follow-up were included in the primary analysis of the study. The LSI was analysed dichotomously where patients achieving an LSI of ≥ 90% in all tests of muscle function were compared with patients not achieving this cut-off value. This cut-off is based on the recommendation from the European Board of Sports Rehabilitation [34] and achieving this cut-off has been reported to decrease the risk of subsequent ACL injury after returning to sport [12, 18]. Secondary analyses were performed for the knee extension and flexion strength tests with all results available from the one-year follow-up. An LSI of ≥ 90% for each test was used as a cut-off for the analyses.

### Statistical analysis

Statistical analysis was performed using the statistical analysis system, SAS System for Windows, version 9 (SAS Institute Inc., Cary, North Carolina, USA). Descriptive statistics for patient demographics and outcomes were reported as numbers and percentages for categorical variables. Continuous variables were reported as the mean, standard deviation, [28] median, first and third quartile. For comparisons between two groups, Fisher’s exact test (lowest one-sided *p* value multiplied by 2) was used for dichotomous variables and the Mann–Whitney *U* test for continuous variables. Binary logistical regression was performed to analyse the association between predictors and recovery of muscle function. For the primary analysis, the recovery of muscle function was defined as achieving an LSI of ≥ 90% in all the tests of muscle function and was used as a dependent variable. The presence of concomitant injuries was used dichotomously (yes/no) as an independent variable in the regression. Graft choice was analysed by comparing hamstring tendon (HT) and patellar tendon (PT) autografts. Patient demographics for gender, age (per 10 years), weight, height, body mass index and pre-injury level of physical activity measured using the Tegner activity score [33] were included as independent variables. The results from the logistic regression models were presented with the odds ratio (OR), 95% confidence intervals (CI) and *p* values. The area under the receiver operating characteristics (ROC) curve was given as a measurement of goodness of fit. In addition, sensitivity analyses were performed by calculating the association of concomitant injuries and the recovery of symmetrical strength in knee extension and flexion, also defined as ≥ 90% in LSI. Finally, in an attempt to find the best predictive model for achieving an LSI of ≥ 90% in all the tests of muscle function, knee extension strength and knee flexion strength, a step-wise multivariable logistic model was used. Multivariable analyses were performed for muscle function tests that had at least one significant outcome in the univariable analyses. Predictors with *p* < 0.20 were entered into the step-wise analyses. All significance tests were two-sided and conducted at the 5% significance level.

## Results

**Fig. 1:**
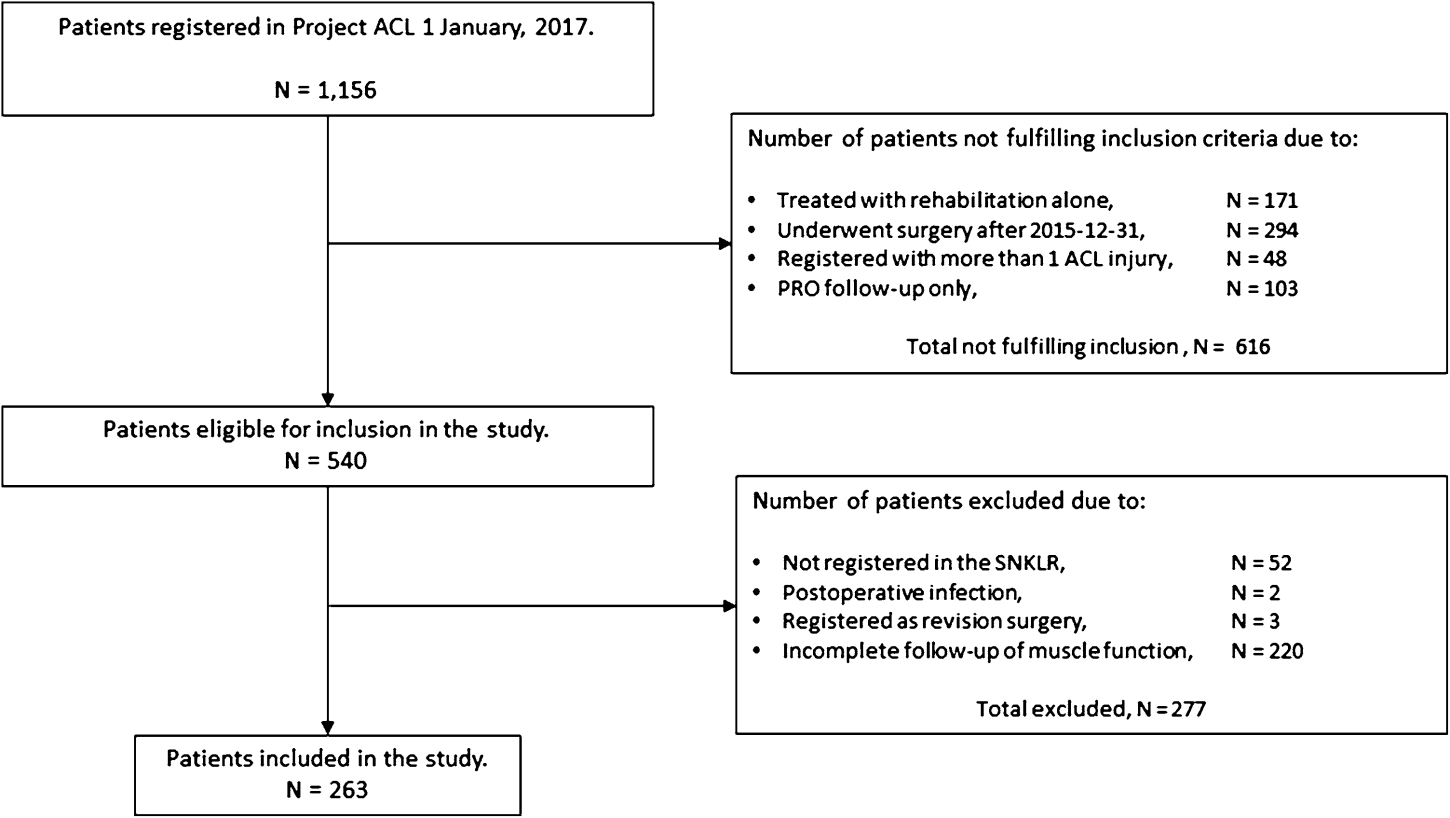
Flow chart of included and excluded PRO; patient-reported outcome

A total of 263 patients fulfilled the inclusion criteria for the study (Fig. 1). Patient sex was evenly distributed in the cohort, 124 patients (47%) were females. The average age at ACL reconstruction among the included patients was 28 ± 10 years. A total of 89% of the cohort received a hamstring tendon autograft and the most common activity that led to ACL injury was football. A meniscus injury was the most common concomitant injury in the cohort (44%). Patient demographics and a drop-out analysis are presented in Table 1. No differences in baseline demographics, the presence of concomitant injuries or the pre-injury level of physical activity were found for included and excluded patients. The results from the 1-year follow-up of muscle function are available in Supplementary Table 1.

**Table 1:** Baseline data and drop-out analysis

	Not recovered (*n* = 166)	Recovered, LSI ≥ 90% in all five tests (*n* = 47)	Total cohort (*n* = 263)	Excluded (*n* = 277)	*p* value
Patient demographics					
Patient sex					
Female	75 (45.2%)	17 (36.2%)	124 (47.1%)	154 (55.6%)	
Male	91 (54.8%)	30 (63.8%)	139 (52.9%)	123 (44.4%)	n.s
Age at index ACL injury	26.1 (9.6) 24.0 (11.5; 54.5) (18.3; 30.5) *n* = 166	25.8 (9.4) 24.1 (13.2; 54.7) (19.7; 28.3) *n* = 47	26.7 (10.3) 24.4 (11.5; 57.7) *n* = 263	26.5 (10.3) 23.3 (9.9; 61.0) *n* = 276	n.s
Age at index ACL reconstruction	27.5 (10.0) 25.9 (12.0; 55.9) (19.8; 33.7) *n* = 166	26.4 (9.4) 24.3 (13.7; 55.0) (20.7; 29.6) *n* = 47	28.0 (10.5) 26.0 (12.0; 58.4) *n* = 263	27.1 (10.3) 23.9 (12.0; 57.3) *n* = 277	n.s
Height (cm)	174.4 (9.4) 175.0 (167.0; 182.0) *n* = 160	177.8 (9.2) 178.0 (170.0; 184.0) *n* = 46	174.8 (9.6) 175.0 (151.0; 200.0) *n* = 263	174.4 (9.1) 174.0 (140.0; 200.0) *n* = 277	n.s
Weight (kg)	74.0 (16.1) 74.0 (64.9; 83.0) *n* = 166	75.1 (15.8) 76.0 (70.0; 81.9) *n* = 47	73.7 (13.0) 73.0 (42.0; 114.0) *n* = 263	73.5 (13.5) 72.0 (35.0; 130.0) *n* = 275	n.s
BMI (kg/m^2^)	24.4 (4.5) 23.8 (22.4; 25.8) *n* = 160	24.2 (2.3) 24.2 (22.5; 25.2) *n* = 46	24.1 (2.8) 23.7 (16.4; 38.7) *n* = 263	24.1 (3.2) 23.6 (17.9; 38.0) *n* = 275	n.s
Surgery-related factors					
Graft choice					
Hamstring tendon	143 (86.7%)	44 (93.6%)	232 (88.9%)	154 (90.1%)	
Patellar tendon	22 (13.3%)	3 (6.4%)	29 (11.1%)	17 (9.9%)	n.s
Concomitant injuries					
Medial meniscus	31 (18.7%)	10 (21.3%)	57 (21.7%)	43 (24.3%)	n.s
Lateral meniscus	47 (28.3%)	14 (29.8%)	72 (27.4%)	53 (29.9%)	n.s
Articular cartilage	41 (24.7%)	15 (31.9%)	75 (27.0%)	47 (26.9%)	n.s
Medial collateral ligament	7 (4.2%)	3 (6.4%)	13 (4.9%)	11 (6.2%)	n.s
Lateral collateral ligament	0 (0.0%)	1 (2.1%)	1 (0.4%)	2 (1.1%)	n.s
Meniscus (medial or lateral)	70 (42.2%)	23 (48.9%)	116 (44.1%)	82 (46.3%)	n.s
Activity					
Football	67 (40.1%)	24 (49.0%)	105 (39.9%)	107 (45.5%)	n.s
Tegner activity level_preop_ ≥ 6	128 (80.0%)	39 (84.8%)	198 (78.0%)	98 (85.2%)	n.s

For categorical variables, *n* (%) is presented

For continuous variables, the mean (SD)/median (Q1; Q3)/*n* = is presented

For comparisons between groups, Fisher’s exact test (lowest one-sided *p* value multiplied by 2) was used for dichotomous variables and the Mann–Whitney *U* test was used for continuous variables

*ACL* anterior cruciate ligament, *BMI* body mass index, *LSI* limb symmetry index

**Fig. 2:**
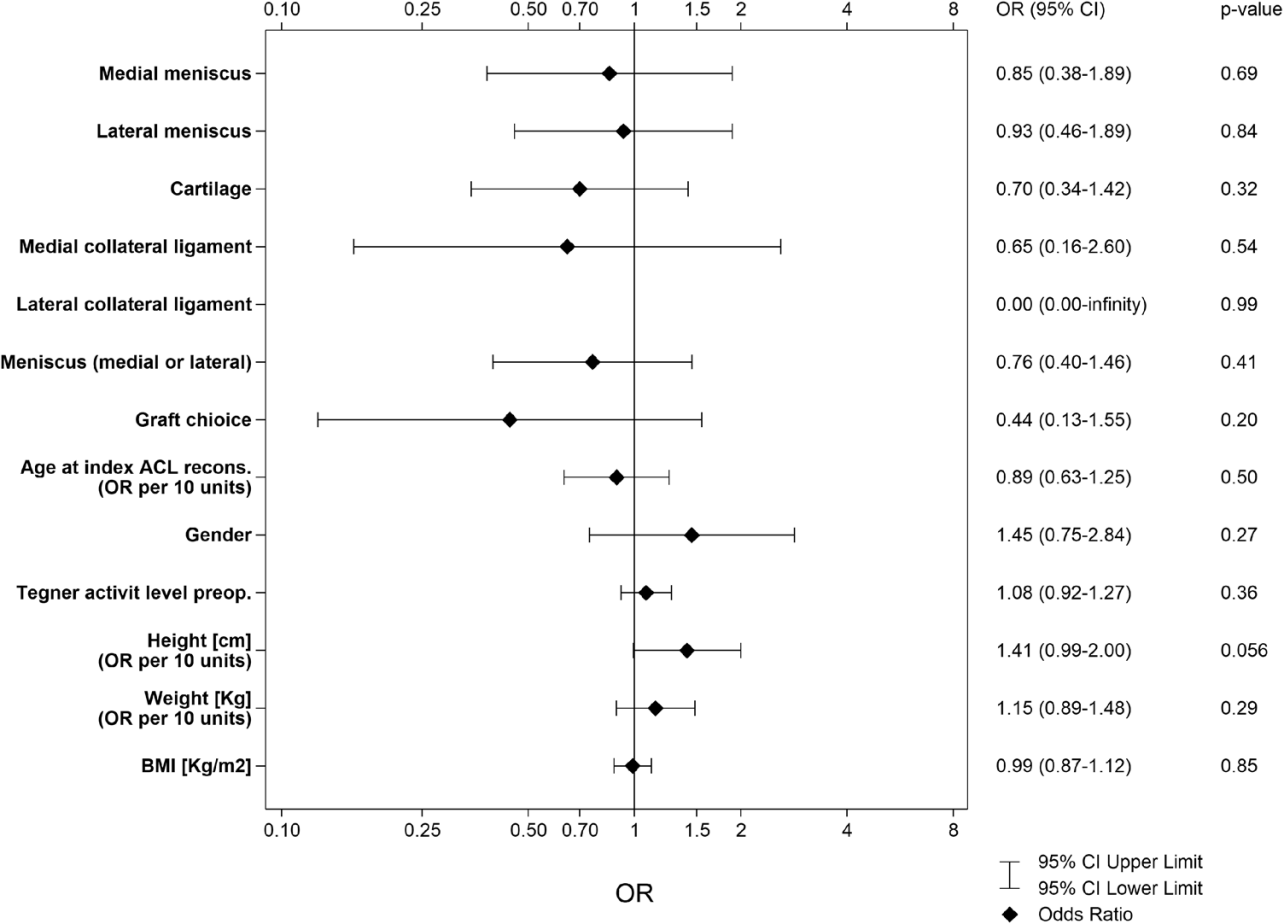
Odds ratio (OR), 95% confidence intervals and area under the curve from the receiver operating characteristic for a limb symmetry index of ≥ 90% in all five tests strength. An OR of > 1 indicates a result favouring the absence of a concomitant injury. For graft choice, an OR of < 1 indicates that the result favours a hamstring tendon autograft, while an OR of > 1 favours a patellar tendon autograft

No patient demographic or intra-operative predictors were found to be statistically significant when attempting to predict achieving an LSI of ≥ 90% in all five tests of muscle function (Table 2; Fig. 2).

**Table 2:** Univariable logistic regression model with a limb symmetry index of ≥ 90% in all five tests of muscle function as dependent outcome

Predictors	*n* missing	Value	Recovered “Yes”	OR (95% CI) muscle recovery (all LSI ≥ 90%)	*p* value	Area under ROC curve (95% CI)
Concomitant injuries						
Medial meniscus	0	Yes	10 (24.4%)			
		No	37 (21.5%)	0.85 (0.38–1.89)	n.s	0.51 (0.45–0.58)
Lateral meniscus	0	Yes	14 (23.0%)			
		No	33 (21.7%)	0.93 (0.46–1.89)	n.s	0.51 (0.43–0.58)
Articular cartilage	0	Yes	15 (26.8%)			
		No	32 (20.4%)	0.70 (0.34–1.42)	n.s	0.54 (0.46–0.61)
Medial collateral ligament	0	Yes	3 (30.0%)			
		No	44 (21.7%)	0.65 (0.16–2.60)	n.s	0.51 (0.47–0.55)
Lateral collateral ligament	0	Yes	1 (100.0%)			
		No	46 (21.7%)	0.00 (0.00–infinity)	n.s	0.51 (0.49–0.53)
Meniscus (medial or lateral)	0	Yes	23 (24.7%)			
		No	24 (20.0%)	0.76 (0.40–1.46)	n.s	0.53 (0.45–0.62)
Surgery-related factors						
Graft choice	7	Hamstring tendon	44 (23.5%)			
		Patellar tendon	3 (12.0%)	0.44 (0.13–1.55)	n.s	0.53 (0.49–0.58)
Patient demographics						
Age at index ACL reconstruction (OR per 10 units)	0	12– < 25	25 (24.3%)			
		25– < 35	15 (21.7%)			
		35–58	7 (17.1%)	0.89 (0.63–1.25)	n.s	0.53 (0.44–0.62)
Patient sex	0	Female	17 (18.5%)			
		Male	30 (24.8%)	1.45 (0.75–2.84)	n.s	0.55 (0.47–0.62)
Tegner activity level pre-operative (0–10)	9	1–6	11 (19.0%)			
		7	9 (27.3%)			
		8	6 (12.8%)			
		9	15 (31.3%)			
		10	5 (25.0%)	1.08 (0.92–1.27)	n.s	0.55 (0.45–0.64)
Height (cm) (OR per 10 units)	0	151– < 170	11 (16.2%)			
		170– < 180	16 (23.5%)			
		180–200	20 (26.0%)	1.41 (0.99–2.00)	n.s	0.58 (0.49–0.67)
Weight (kg) (OR per 10 units)	0	42– < 67	13 (18.3%)			
		67– < 79	19 (26.0%)			
		79–114	15 (21.7%)	1.15 (0.89–1.48)	n.s	0.54 (0.45–0.63)
BMI (kg/m^2^)	0	16– < 23	23 (26.4%)			
		23– < 25	9 (14.1%)			
		25–39	15 (24.2%)	0.99 (0.87–1.12)	n.s	0.52 (0.43–0.61)

“Yes/No” indicates the presence of the described concomitant injury

All tests were performed with univariable logistic regression

*p* values, OR and area under ROC curve were based on original values and not on stratified groups

OR is the ratio for the odds of an increase in the predictor of one unit

*ACL* anterior cruciate ligament, *OR* odds ratio, *CI* confidence interval, *ROC* receiver operating characteristic

In the univariable analysis aiming to predict the achievement of an LSI ≥ 90% in knee extension strength, the absence of a lateral meniscus (LM) injury was associated with decreased OR = 0.51 [(95% CI 0.27–0.99), *p* = 0.048]. In addition, a PT autograft and older age decreased the odds of achieving an LSI of ≥ 90% in knee extension strength, OR = 0.32 [(95% CI 0.15–0.70), *p* = 0.0043] and OR = 0.76 [(0.60–0.98), *p* = 0.034] respectively. The absence of a concomitant LM injury, PT autograft and older age were also found to decrease the odds in the multivariable analysis, OR = 0.51 [(95% CI 0.25–0.99), *p* = 0.048], OR = 0.24 [(95% CI 0.10–0.55), *p* = 0.0008] and OR = 0.73 [(95% CI 0.56–0.94), *p* = 0.017] (Table 3 and Supplementary Tables 2 and 3). A higher pre-injury level of physical activity was the only predictor found to be significant when attempting to predict achieving an LSI of ≥ 90% in knee flexion strength, OR = 1.14 [(95% CI 1.01–1.29), *p* = 0.037] (Table 4 and Supplementary Tables 4 and 5).

**Table 3:** Univariable and multivariable logistic regression model with a limb symmetry index of ≥ 90% in the knee extension test as dependent outcome

Predictors	*n* missing	Value	Recovered “Yes”	Univariable*			Multivariable***	
				OR (95% CI) LSI knee extension ≥ 90%	*p* value	Area under ROC curve (95% CI)	OR (95% CI) LSI knee extension ≥ 90%	*p* value
Concomitant injuries								
Medial meniscus	0	Yes	37 (64.9%)					
		No	151 (73.3%)	1.48 (0.79–2.77)	n.s	0.53 (0.48–0.59)		
Lateral meniscus	0	Yes	58 (80.6%)					
		No	130 (68.1%)	0.51 (0.27–0.99)	0.048	0.56 (0.51–0.62)	0.50 (0.25–0.99)	0.049
Articular cartilage	0	Yes	55 (73.3%)					
		No	133 (70.7%)	0.88 (0.48–1.60)	n.s	0.51 (0.45–0.57)		
Medial collateral ligament	0	Yes	7 (53.8%)					
		No	181 (72.4%)	2.25 (0.73–6.93)	n.s	0.52 (0.49–0.56)		
Lateral collateral ligament	0	Yes	1 (100.0%)					
		No	187 (71.4%)	0.00 (0.00–infinity)	n.s	0.50 (0.50–0.51)		
Meniscus (medial or lateral)	0	Yes	86 (74.1%)					
		No	102 (69.4%)	0.79 (0.46–1.36)	n.s	0.53 (0.46–0.59)		
Surgery-related factors								
Graft choice	7	Hamstring tendon	173 (74.6%)					
		Patella tendon	14 (48.3%)	0.32 (0.15–0.70)	0.0043	0.56 (0.51–0.61)	0.25 (0.11–0.57)	0.0033
Patient demographics								
Age at index ACL reconstruction (OR per 10 units)	0	12– < 25	98 (82.4%)					
		25– < 35	53 (62.4%)					
		35–58	37 (62.7%)	0.76 (0.60–0.98)	0.034	0.60 (0.52–0.67)	0.73 (0.56–0.94)	0.017
Patient sex	0	Female	86 (69.4%)					
		Male	102 (73.4%)	1.22 (0.71–2.08)	n.s	0.52 (0.46–0.59)		
Tegner activity scale pre-operative (0–10)	9	1–6	51 (63.0%)					
		7	29 (70.7%)					
		8	41 (74.5%)					
		9	38 (73.1%)					
		10	21 (84.0%)	1.13 (1.00–1.28)	n.s	0.58 (0.51–0.66)		
Height (cm) (OR per 10 units)	0	151– < 170	56 (64.4%)					
		170– < 180	71 (83.5%)					
		180–200	61 (67.0%)	1.05 (0.80–1.39)	n.s	0.51 (0.42–0.59)		
Weight (kg) (OR per 10 units)	0	42– < 67	63 (70.8%)					
		67– < 79	67 (77.9%)					
		79–114	58 (65.9%)	1.00 (0.82–1.23)	n.s	0.51 (0.42–0.59)		
BMI (kg/m^2^)		16– < 23	73 (70.9%)					
		23– < 25	57 (70.4%)					
		25–39	58 (73.4%)	1.00 (0.91–1.10)	n.s	0.49 (0.41–0.57)		

“Yes/No” indicates the presence of the described concomitant injury

*p* values, OR and area under ROC curve are based on original values and not on stratified groups

OR is the ratio for the odds of an increase in the predictor of one unit

Area under ROC curve with 95% CI for multivariable model = 0.62 (0.55–0.68)

*ACL* anterior cruciate ligament, *OR* odds ratio, *CI* confidence interval, *ROC* receiver operating characteristic

*All tests were performed with univariable logistic regression

***Multivariable logistic regression model including graft choice and lateral meniscus

**Table 4:** Univariable logistic regression model with a limb symmetry index of ≥ 90% in the knee flexion test as dependent outcome

Predictors	*n* missing	Value	Recovered “Yes”	Univariable*		
				OR (95%CI) LSI knee flexion ≥ 90%	*p* value	Area under ROC curve (95%CI)
Concomitant injuries						
Medial meniscus	0	Yes	38 (66.7%)			
		No	148 (71.8%)	1.28 (0.68–2.39)	n.s	0.52 (0.46–0.58)
Lateral meniscus	0	Yes	47 (65.3%)			
		No	139 (72.8%)	1.42 (0.80–2.54)	n.s	0.54 (0.47–0.60)
Articular cartilage	0	Yes	55 (73.3%)			
		No	131 (69.7%)	0.84 (0.46–1.52)	n.s	0.52 (0.46–0.58)
Medial collateral ligament	0	Yes	10 (76.9%)			
		No	176 (70.4%)	0.71 (0.19–2.67)	n.s	0.51 (0.48–0.53)
Lateral collateral ligament	0	Yes	1 (100.0%)			
		No	185 (70.6%)	0.00 (0.00–infinity)	n.s	0.50 (0.50–0.51)
Meniscus (medial or lateral)	0	Yes	77 (66.4%)			
		No	109 (74.1%)	1.45 (0.85–2.48)	n.s	0.55 (0.48–0.61)
Surgery-related factors						
Graft choice	7	Hamstring tendon	160 (69.0%)			
		Patellar tendon	25 (86.2%)	2.81 (0.94–8.38)	n.s	0.54 (0.51–0.58)
Patient demographics						
Age at index ACL reconstruction (OR per 10 units)	0	12– < 25	87 (73.1%)			
		25– < 35	63 (74.1%)			
		35–58	36 (61.0%)	0.87 (0.68–1.11)	n.s	0.53 (0.45–0.61)
Patient sex	0	Female	81 (65.3%)			
		Male	105 (75.5%)	1.64 (0.96–2.80)	n.s	0.56 (0.50–0.63)
Tegner activity scale pre-operative (0–10)	34	1–6	51 (63.0%)			
		7	32 (78.0%)			
		8	36 (65.5%)			
		9	41 (78.8%)			
		10	21 (84.0%)	1.14 (1.01–1.29)	0.037	0.59 (0.51–0.66)
Height (cm) (OR per 10 units)	0	151– < 170	57 (65.5%)			
		170– < 180	61 (71.8%)			
		180–200	68 (74.7%)	1.22 (0.92–1.62)	n.s	0.55 (0.47–0.63)
Weight (kg) (OR per 10 units)	0	42– < 67	55 (61.8%)			
		67– < 79	66 (76.7%)			
		79–114	65 (73.9%)	1.19 (0.96–1.47)	n.s	0.57 (0.49–0.64)
BMI (kg/m^2^)	0	16– < 23	70 (68.0%)			
		23– < 25	58 (71.6%)			
		25–39	58 (73.4%)	1.06 (0.96–1.17)	n.s	0.54 (0.46–0.61)

“Yes/No” indicates the presence of the described concomitant injury

*p* values, OR and area under ROC curve are based on original values and not on stratified groups

OR is the ratio for the odds of an increase in the predictor of one unit

*ACL* anterior cruciate ligament, *OR* odds ratio, *CI* confidence interval, *ROC* receiver operating curve

*All tests are performed with univariable logistic regression

## Discussion

The primary finding in the present study was that patients with and without concomitant intra-articular injuries had comparable odds of achieving an LSI of ≥ 90% in five tests of muscle function one year after ACL reconstruction. It is noteworthy that only 23% of patients achieved symmetrical function in all the performed tests. In the analysis of knee extension strength, patients who received an HT autograft and had an LM injury had favourable odds of achieving symmetrical strength. In addition, a small favourable effect was found for younger age. However, it is important to remember that all the logistic regression models resulted at best in a poor goodness of fit, which indicates that other factors that were not included in the analyses contribute to achieving a symmetrical performance across a battery of tests.

Remarkably, over 75% of patients were not able to achieve symmetrical results in all five tests of muscle function one year after surgery (Table 1 and Supplementary Table 1). This confirms previous findings in the scientific literature and implies that restoring muscular capacity may take longer than the 9–12 months that is commonly used as a reference [7, 11, 35, 38, 41] and therefore may have important indications for RTS [12].

The presence of concomitant injuries was not associated with the recovery of muscle function in this study, which can be due to that one year is a sufficient timeframe to recover from the short-term impairment caused by concomitant injuries or due to the limited sensory innervation of these structures misleading the perceptions of the severity of these injuries [4, 5, 10, 22, 39]. However, it is well known that the presence of a concomitant injury is associated with a long-term risk of impaired knee function [27]. Degenerative changes in joints, such as osteoarthritis, take time to develop and it is currently not known if the risk of these changes or their impairment can be reduced by achieving symmetrical knee function [30]. The lack of association found in this study questions whether other factors are equally or more important to include obtaining a complete understanding of what affects the recovery of muscle function after ACL reconstruction, e.g. psychological characteristics and pre-operative or early post-operative knee function. However, the lack of an association between concomitant injuries and the recovery of muscle function may also be contributed to that achieving a symmetrical muscle function across a battery of tests is a difficult short-term goal to attain after ACL reconstruction [34], nonetheless, reflected by the low proportion of patients reaching this benchmark in the present study. It is, therefore, suggested that the results of this study should be taken into consideration when planning rehabilitation programmes and further emphasis should be placed on ensuring that compliance and intensity levels in training are kept appropriate to improve the recovery of muscle function after ACL reconstruction.

The LSI is one of the most common ways of presenting the outcome of tests of muscle function, as it facilitates an understanding of the results [7, 25]. However, there is a possible overestimation of results when using measurements of limb symmetry to evaluate the recovery of knee function, even when rigorous RTS criteria are used, i.e. an LSI of ≥ 90% in multiple tests or the recovery of pre-injury status [7, 11, 38]. Wellstandt et al. [38] evaluated the uninvolved limb as a reference standard for symmetry indices used in RTS testing. The authors found that estimated pre-injury levels of muscular strength and performance, based on tests conducted at an early stage after ACL injury, may be a better reference for the recovery of muscle function, considering the decrease in strength in the uninvolved limb that usually occurs during rehabilitation [38]. Over time, this reduction in the function of the uninvolved leg will inflate the LSIs and lead to a misrepresentation of the functional ability of the injured limb [11, 38]. In addition, it should be pointed out that the LSI ratio is based on two independent tests, one on each limb, with their own variability. This makes the use of the LSI subject to uncertain variability which can over- or underestimate the true discrepancy between the patient’s limbs. Not fulfilling the RTS criteria in five strength and hop tests has, which is of great clinical importance, been associated with a large increase in the risk of knee re-injury [21, 34]. Therefore, achieving symmetrical knee function after ACL reconstruction should be regarded as a fundamental goal of rehabilitation with regard to the protective effects of secondary injuries. However, it remains to be established whether this goal is sufficient to ensure a safe RTS and limit long-term impairments.

Graft choice between PT and HT autografts did not influence the likelihood of achieving symmetrical knee function. However, it should be noted that only a small proportion of patients (*n* = 25) received PT autografts, which induces uncertainty in the regression models, reflected by the wide confidence interval. Nevertheless, graft selection in ACL reconstruction is always accompanied by donor site morbidity impairing muscle function from the harvest site and creates morphological changes [16, 35]. In particular, patients undergoing ACL reconstruction with PT autograft have been reported to have cellular alternations of the quadriceps, which may explain the difficulties in restoring knee extension strength in the short term [24, 35, 40]. This finding was consistent in both the univariable and multivariable analyses of the present study. In addition, favourable odds of achieving symmetrical knee extension strength were found in the presence of LM injury at ACL reconstruction, but this finding should be interpreted with caution. A meniscal injury is usually associated with pain and the limitation of the patient’s knee joint [15] and, therefore, there is no reason to believe that the presence of such injury has a favourable effect on the recovery of muscle function [10, 20]. There is a possibility that patients who sustained a concomitant LM injury underwent a different rehabilitation regime, with altered timelines and delayed onset of full weight bearing and strength training, but no data are kept on these variables in the registers. In the sensitivity analysis performed on the knee extension test, the favourable odds of achieving symmetrical performance were only found in patients who were evaluated with the isometric test. Only a small proportion of patients were evaluated with the isometric test, which may have biased the analysis and could partly explain the significant effect from the presence of LM injury. In addition, no association was found between meniscal injury and the recovery of symmetrical knee extension strength, when both medial and LM injuries were included.

## Limitations

Pre-operative results of muscle function were not available for all the included patients, which may pose a limitation, as strength differences in patients with and without concomitant injuries may have been present before they were treated with reconstructive surgery. The presence of concomitant injures was analysed dichotomously at reconstruction and no attention was, therefore, paid to the potential differences in the surgical and rehabilitation treatment. Moreover, the lack of data on the size and severity of concomitant injuries may mean that the dichotomous analysis of concomitant injuries was not sensitive enough to identify differences in the recovery of muscle function and could act as a limitation in the study. In addition, a very small proportion of patients had an injury to either the medial (*n* = 18) or the lateral (*n* = 1) collateral ligaments which limits the ability to draw conclusions related to these injuries. Finally, the low values of the ROC curve analyses suggested that none of the predictors in the study can be regarded as strong, despite their potential influence on clinical practice.

To our knowledge, this is the first study to examine the effect of concomitant knee injuries present during ACL reconstruction on the recovery of muscle function one year after surgery. This study provides unique data, comprising detailed information and clinical outcome from physiotherapists and surgeons, in a large cohort of patients.

## Conclusion

No negative effect on the short-term recovery of symmetrical performance in five tests of muscle function was found in the presence of intra-operatively identified concomitant injuries; in the present study, comprising data on patients who had undergone ACL reconstruction from two registers. However, fewer than one in four patients in the total cohort achieved an LSI of ≥ 90% across the battery of tests, which may have implications for RTS.

In addition, younger age and HT autografts were favourable in terms of the recovery of knee extension strength, but graft choice did not influence the possibility of symmetrical performance across all five tests of muscle function one year after ACL reconstruction.

## Electronic supplementary material

Below is the link to the electronic supplementary material.

Supplementary material 1 (DOCX 19 KB)

Supplementary material 2 (DOCX 27 KB)
