# Supplementary material for: Concomitant injuries may not reduce the likelihood of achieving symmetrical muscle function one year after anterior cruciate ligament reconstruction: a prospective observational study based on 263 patients
Source: Knee Surg Sports Traumatol Arthrosc. 2018 Feb 5;26(10):2966–77. doi: 10.1007/s00167-018-4845-2 (PMC6154030; doi:10.1007/s00167-018-4845-2)
Supplement: Supplementary file 1 — Supplementary material 1 (DOCX 19 KB) [file 167_2018_4845_MOESM1_ESM.docx]

| **Appendix table A1. Twelve-month outcomes.** | |
| --- | --- |
|  | **Included (n = 263)** |
| **Activity** | |
| Tegner activity scale 12 months [0-10] | 5.6 (2.4) 5.0 (4.0; 8.0) n = 263 |
| Change in Tegner activity scale from pre-injury to 12-month follow-up |  |
| Reduced | 59.5% |
| Equal | 28.2% |
| Improved | 12.4% |
| Returned to knee demanding sport, i.e. Tegner activity level ≥6 |  |
| No | 43.0% |
| Yes | 57.0% |
| Change in Tegner activity scale from pre-Injury to follow-up (among Tegner_preop_ ≥6) |  |
| Reduced | 67.6% |
| Equal | 26.1% |
| Improved | 6.3% |
| **Muscular function - Limb Symmetri Indices (LSI)** | |
| LSI Knee Extensor [%] | 94.4 (12.7) 95.4 (89.1; 101.0) n = 263 |
| LSI Knee Extensor [%] (isometric) | 94.1 (12.6) 95.5 (89.8; 102.2) n = 91 |
| LSI Knee Extensor [%] (isokinetic) | 94.6 (12.8) 95.3 (89.0; 100.7) n = 172 |
| LSI Knee Flexor [%] | 96.2 (14.2) 96.4 (87.6; 104.4) n = 263 |
| LSI Knee Flexor [%] (isometric) | 93.1 (16.4) 93.6 (84.8; 103.4) n = 91 |
| LSI Knee Flexor [%] (isokinetic) | 97.9 (12.6) 97.4 (90.1; 104.5) n = 172 |
| LSI Vertical Jump [%] | 89.2 (16.0) 89.3 (79.8; 99.1) n = 242 |
| LSI Hop for Distance [%] | 94.1 (10.1) 95.0 (89.7; 100.7) n = 238 |
| LSI Crossover Hops [%] | 94.4 (16.9) 96.2 (86.1; 103.9) n = 213 |
| **Muscle function - Female** | |
| **Absolute and relative results of injured leg** | |
| Knee Extension [Nm] (isometric) | 173.3 (45.9) 169.0 (147.0; 197.0) n = 49 |
| Knee Extension [Nm] (isokinetic) | 147.6 (31.2) 148.0 (128.0; 169.0) n = 75 |
| Knee Flexion [Nm] (isometric) | 100.5 (29.9) 98.0 (80.0; 126.0) n = 49 |
| Knee Flexion [Nm] (isokinetic) | 82.2 (18.0) 83.0 (73.0; 91.0) n = 75 |
| Vertical Jump [cm] | 10.3 (4.8) 10.8 (7.2; 13.4) n = 124 |
| Hop for Distance [cm] | 95.9 (42.4) 106.5 (78.0; 127.0) n = 124 |
| Side hop [n] | 27.4 (21.0) 27.0 (1; 49) n = 124 |
| Relative strength Knee Extension [Nm/Kg] (isometric) | 2.56 (0.66) 2.50 (2.23; 2.99) n = 49 |
| Relative strength Knee Extension [Nm/Kg] (isokinetic) | 2.23 (0.44) 2.28 (1.90; 2.56) n = 73 |
| Relative strength Knee Flexion [Nm/Kg] (isometric) | 1.49 (0.46) 1.44 (1.23; 1.83) n = 49 |
| Relative strength Knee Flexion [Nm/Kg] (isokinetic) | 1.24 (0.25) 1.25 (1.09; 1.37) n = 73 |
| Relative Vertical Jump [cm/Kg] | 0.17 (0.07) 0.17 (0.13; 0.22) n = 112 |
| Relative Strength Hop for Distance [cm/Kg] | 1.64 (0.48) 1.65 (1.35; 1.99) n = 108 |
| Relative Side Hop [n/Kg] | 0.57 (0.25) 0.50 (0.35; 0.81) n = 90 |
| **Absolute and relative results of uninvolved leg** | |
| Knee Extension [Nm] (isometric) | 187.6 (36.5) 190.0 (161.0; 213.0) n = 49 |
| Knee Extension [Nm] (isokinetic) | 158.5 (27.8) 155.0 (141.0; 174.0) n = 75 |
| Knee Flexion [Nm] (isometric) | 110.8 (23.0) 112.0 (95.0; 127.0) n = 49 |
| Knee Flexion [Nm] (isokinetic) | 84.6 (16.7) 84.0 (74.0; 98.0) n = 75 |
| Vertical Jump [cm] | 11.7 (5.1) 12.3 (9.2; 14.9) n = 124 |
| Hop for Distance [cm] | 101.7 (42.6) 113.0 (90.0; 129.0) n = 124 |
| Side hop [n] | 30.2 (22.3) 29.5 (0.0; 50.5) n = 124 |
| Relative strength Knee Extension [Nm/Kg] (isometric) | 2.77 (0.56) 2.78 (2.43; 3.10) n = 49 |
| Relative strength Knee Extension [Nm/Kg] (isokinetic) | 2.41 (0.41) 2.40 (2.17; 2.69) n = 73 |
| Relative strength Knee Flexion [Nm/Kg] (isometric) | 1.64 (0.38) 1.64 (1.39; 1.83) n = 49 |
| Relative strength Knee Flexion [Nm/Kg] (isokinetic) | 1.28 (0.22) 1.28 (1.16; 1.43) n = 73 |
| Relative Vertical Jump [cm/Kg] | 0.19 (0.07) 0.19 (0.15; 0.24) n = 112 |
| Relative Strength Hop for Distance [cm/Kg] | 1.74 (0.46) 1.80 (1.48; 2.04) n = 108 |
| Relative Side Hop [n/Kg] | 0.63 (0.26) 0.63 (0.40; 0.84) n = 90 |
| **Muscle function - Men** | |
| **Absolute and relative results of injured leg** | |
| Knee Extension [Nm] (isometric) | 280.3 (60.6) 280.5 (256.0; 314.0) n = 42 |
| Knee Extension [Nm] (isokinetic) | 230.3 (41.7) 233.0 (197.0; 259.0) n = 97 |
| Knee Flexion [Nm] (isometric) | 167.4 (43.4) 170.5 (139.0; 199.0) n = 42 |
| Knee Flexion [Nm] (isokinetic) | 129.3 (24.1) 131.0 (113.0; 144.0) n = 97 |
| Vertical Jump [cm] | 15.2 (6.2) 15.9 (11.9; 19.1) n = 139 |
| Hop for Distance [cm] | 126.8 (43.7) 137.0 (115.0; 156.0) n = 139 |
| Side hop [n] | 43.8 (22.0) 48.0 (31.0; 62.0) n = 139 |
| Relative strength Knee Extension [Nm/Kg] (isometric) | 3.51 (0.64) 3.50 (3.14; 3.84) n = 42 |
| Relative strength Knee Extension [Nm/Kg] (isokinetic) | 2.80 (0.48) 2.78 (2.48; 3.09) n = 96 |
| Relative strength Knee Flexion [Nm/Kg] (isometric) | 2.09 (0.47) 2.18 (1.78; 2.37) n = 42 |
| Relative strength Knee Flexion [Nm/Kg] (isokinetic) | 1.57 (0.30) 1.51 (1.38; 1.75) n = 96 |
| Relative Vertical Jump [cm/Kg] | 0.20 (0.07) 0.20 (0.15; 0.24) n = 128 |
| Relative Strength Hop for Distance [cm/Kg] | 1.70 (0.42) 1.72 (1.43; 1.95) n = 128 |
| Relative Side Hop [n/Kg] | 0.63 (0.23) 0.64 (0.50; 0.77) n = 121 |
| **Absolute and relative results of uninvolved leg** | |
| Knee Extension [Nm] (isometric) | 291.0 (57.5) 296.0 (252.0; 330.0) n = 42 |
| Knee Extension [Nm] (isokinetic) | 242.6 (40.6) 240.0 (216.0; 261.0) n = 97 |
| Knee Flexion [Nm] (isometric) | 173.7 (37.4) 176.5 (147.0; 195.0) n = 42 |
| Knee Flexion [Nm] (isokinetic) | 131.9 (20.7) 135.0 (117.0; 145.0) n = 97 |
| Vertical Jump [cm] | 16.7 (6.3) 17.8 (14.0; 20.4) n = 138 |
| Hop for Distance [cm] | 133.2 (45.2) 145.0 (124.0; 160.0) n = 139 |
| Side hop [n] | 44.5 (21.9) 51.0 (33.0; 61.0) n = 139 |
| Relative strength Knee Extension [Nm/Kg] (isometric) | 3.65 (0.60) 3.72 (3.36; 3.99) n = 42 |
| Relative strength Knee Extension [Nm/Kg] (isokinetic) | 2.94 (0.42) 2.91 (2.66; 3.23) n = 96 |
| Relative strength Knee Flexion [Nm/Kg] (isometric) | 2.18 (0.41) 2.17 (1.85; 2.42) n = 42 |
| Relative strength Knee Flexion [Nm/Kg] (isokinetic) | 1.60 (0.22) 1.57 (1.46; 1.73) n = 96 |
| Relative Vertical Jump [cm/Kg] | 0.23 (0.07) 0.23 (0.18; 0.26) n = 127 |
| Relative Strength Hop for Distance [cm/Kg] | 1.80 (0.39) 1.84 (1.53; 2.05) n = 127 |
| Relative Side Hop [n/Kg] | 0.65 (0.21) 0.66 (0.53; 0.78) n = 120 |
| For categorical variables n (%) is presented. For continuous variables Mean (SD) / Median (Q1; Q3) / n= is presented. For comparison between groups Fisher´s Exact test (lowest 1-sided p-value multiplied by 2) was used for dichotomous variables and the Mann-Whitney U-test was used for continuous variables. | |
