# Supplementary material for: Concomitant injuries may not reduce the likelihood of achieving symmetrical muscle function one year after anterior cruciate ligament reconstruction: a prospective observational study based on 263 patients
Source: Knee Surg Sports Traumatol Arthrosc. 2018 Feb 5;26(10):2966–77. doi: 10.1007/s00167-018-4845-2 (PMC6154030; doi:10.1007/s00167-018-4845-2)
Supplement: Supplementary file 2 — Supplementary material 2 (DOCX 27 KB) [file 167_2018_4845_MOESM2_ESM.docx]

| **Appendix table A2. Univariable logistic regression model with limb symmetry index ≥ 90% in isometric knee extension strength as dependent outcome.** | | | | | | |
| --- | --- | --- | --- | --- | --- | --- |
| **Predictors** | **n missing** | **Value** | **Reconvered”yes”** | **OR (95% CI) LSI Knee Extension ≥90%** | **p-value** | **Area under ROC-Curve (95% CI)** |
| **Concomitant injuries** | | | | | | |
| Medial meniscus | 0 | Yes | 15 (78.9%) |  |  |  |
|  |  | No | 53 (73.6%) | 0.74 (0.22-2.52) | 0.63 | 0.52 (0.43-0.62) |
| Lateral meniscus | 0 | Yes | 21 (91.3%) |  |  |  |
|  |  | No | 47 (69.1%) | 0.21 (0.05-0.99) | 0.049 | 0.61 (0.53-0.69) |
| Articular cartilage | 0 | Yes | 15 (75.0%) |  |  |  |
|  |  | No | 53 (74.6%) | 0.98 (0.31-3.08) | 0.97 | 0.50 (0.40-0.60) |
| Medial collateral ligament | 0 | Yes | 2 (50.0%) |  |  |  |
|  |  | No | 66 (75.9%) | 3.14 (0.42-23.70) | 0.27 | 0.53 (0.47-0.59) |
| Lateral collateral ligament | 0 | Yes | 0 (.%) |  |  |  |
|  |  | No | 68 (74.7%) | 0.00 (0.00-infinity) | . | 0.50 (0.50-0.50) |
| Meniscus (medial or lateral) | 0 | Yes | 32 (84.2%) |  |  |  |
|  |  | No | 36 (67.9%) | 0.40 (0.14-1.13) | 0.083 | 0.60 (0.50-0.71) |
| **Surgery-related factors** | | | | | | |
| Graft choice | 7 | Hamstrings tendon | 63 (75.0%) |  |  |  |
|  |  | Patella tendon | 5 (71.4%) | 0.83 (0.15-4.62) | 0.83 | 0.51 (0.44-0.57) |
| **Anthropometrics** | | | | | | |
| Age at index ACL reconstruction (OR per 10 units) | 0 | 12-<25 | 34 (85.0%) |  |  |  |
|  |  | 25-<35 | 20 (62.5%) |  |  |  |
|  |  | 35-58 | 14 (73.7%) | 0.86 (0.56-1.32) | 0.48 | 0.58 (0.45-0.71) |
| Gender | 0 | Female | 36 (73.5%) |  |  |  |
|  |  | Male | 32 (76.2%) | 1.16 (0.45-2.99) | 0.77 | 0.52 (0.40-0.64) |
| Tegner activity scale preoperative [0-10] | 9 | 1-5 | 7 (58.3%) |  |  |  |
|  |  | 6-10 | 56 (75.7%) | 1.15 (0.90-1.47) | 0.25 | 0.58 (0.44-0.72) |
| ACL; anterior cruciate ligament; OR, Odds ratio; CI, confidence interval; ROC; receiver operating curve.  All tests are performed with univariable logistic regression. P-values, OR and Area under ROC-curve are based on original values and not on stratified groups. OR is the ratio for the odds for an increase of the predictor of one unit. | | | | | | |

| \| **Appendix table A3. Univariable logistic regression model with limb symmetry index ≥ 90% in isometric knee flexion strength as dependent outcome.** \| \| --- \| | | | | | | |
| --- | --- | --- | --- | --- | --- | --- | --- |
| **Predictors** | **n missing** | **Value** | **Recovered ”yes”** | **OR (95% CI) LSI Knee Flexion ≥90%** | **p-value** | **Area under ROC-Curve (95% CI)** |
| **Concomitant injuries** | | | | | | |
| Medial meniscus | 0 | Yes | 12 (63.2%) |  |  |  |
|  |  | No | 44 (61.1%) | 0.92 (0.32-2.61) | 0.87 | 0.51 (0.42-0.59) |
| Lateral meniscus | 0 | Yes | 11 (47.8%) |  |  |  |
|  |  | No | 45 (66.2%) | 2.13 (0.82-5.57) | 0.12 | 0.57 (0.48-0.67) |
| Articular cartilage | 0 | Yes | 14 (70.0%) |  |  |  |
|  |  | No | 42 (59.2%) | 0.62 (0.21-1.80) | 0.38 | 0.54 (0.45-0.62) |
| Medial collateral ligament | 0 | Yes | 4 (100.0%) |  |  |  |
|  |  | No | 52 (59.8%) | 0.00 (0.00-infinity) | 0.27 | N/A |
| Lateral collateral ligament | 0 | Yes | 0 (.%) |  |  |  |
|  |  | No | 56 (61.5%) | 0.00 (0.00-infinity) | . | 0.50 (0.50-0.50) |
| Meniscus (medial or lateral) | 0 | Yes | 21 (55.3%) |  |  |  |
|  |  | No | 35 (66.0%) | 1.57 (0.67-3.70) | 0.30 | 0.56 (0.45-0.66) |
| **Surgery-related factors** | | | | | | |
| Graft choice | 7 | Hamstrings tendon | 51 (60.7%) |  |  |  |
|  |  | Patella tendon | 5 (71.4%) | 1.62 (0.30-8.83) | 0.58 | 0.52 (0.46-0.57) |
| **Anthropometrics** | | | | | | |
| Age at index ACL reconstruction (OR per 10 units) | 0 | 12-<25 | 28 (70.0%) |  |  |  |
|  |  | 25-<35 | 18 (56.3%) |  |  |  |
|  |  | 35-58 | 10 (52.6%) | 0.86 (0.58-1.27) | 0.45 | 0.57 (0.45-0.69) |
| Gender | 0 | Female | 27 (55.1%) |  |  |  |
|  |  | Male | 29 (69.0%) | 1.82 (0.77-4.31) | 0.17 | 0.57 (0.47-0.68) |
| Tegner activity scale preoperative [0-10] | 9 | 1-5 | 6 (50.0%) |  |  |  |
|  |  | 6-10 | 47 (63.5%) | 1.14 (0.91-1.44) | 0.26 | 0.57 (0.45-0.70) |
| ACL; anterior cruciate ligament; OR, Odds ratio; CI, confidence interval; ROC; receiver operating curve.  All tests are performed with univariable logistic regression. P-values, OR and Area under ROC-curve are based on original values and not on stratified groups. OR is the ratio for the odds for an increase of the predictor of one unit. | | | | | | |

| **Appendix table A4. Univariable logistic regression model with limb symmetry index ≥ 90% in isokinetic knee extension strength as dependent outcome.** | | | | | | |
| --- | --- | --- | --- | --- | --- | --- |
| **Predictors** | **n missing** | **Value** | **Recovered ”yes”** | **OR (95% CI) LSI Knee Extension ≥90%** | **p-value** | **Area under ROC-Curve (95%CI)** |
| **Concomitant injuries** | | | | | | |
| Medial meniscus | 0 | Yes | 22 (57.9%) |  |  |  |
|  |  | No | 98 (73.1%) | 1.98 (0.94-4.19) | 0.074 | 0.56 (0.49-0.63) |
| Lateral meniscus | 0 | Yes | 37 (75.5%) |  |  |  |
|  |  | No | 83 (67.5%) | 0.67 (0.32-1.43) | 0.30 | 0.54 (0.47-0.61) |
| Cartilage | 0 | Yes | 40 (72.7%) |  |  |  |
|  |  | No | 80 (68.4%) | 0.81 (0.40-1.65) | 0.56 | 0.52 (0.45-0.60) |
| Medial collateral ligament | 0 | Yes | 5 (55.6%) |  |  |  |
|  |  | No | 115 (70.6%) | 1.92 (0.49-7.45) | 0.35 | 0.52 (0.48-0.56) |
| Lateral collateral ligament | 0 | Yes | 1 (100.0%) |  |  |  |
|  |  | No | 119 (69.6%) | 0.00 (0.00-infinity) | 0.99 | 0.50 (0.50-0.51) |
| Meniscus (medial or lateral) | 0 | Yes | 54 (69.2%) |  |  |  |
|  |  | No | 66 (70.2%) | 1.05 (0.55-2.01) | 0.89 | 0.51 (0.42-0.59) |
| **Surgery-related factors** | | | | | | |
| Graft choice | 7 | Hamstring tendon | 110 (74.3%) |  |  |  |
|  |  | Patella tendon | 9 (40.9%) | 0.24 (0.09-0.60) | 0.0025 | 0.59 (0.52-0.65) |
| **Anthropometrics** | | | | | | |
| Age at index ACL reconstruction (OR per 10 units) | 0 | 12-<25 | 64 (81.0%) |  |  |  |
|  |  | 25-<35 | 33 (62.3%) |  |  |  |
|  |  | 35-58 | 23 (57.5%) | 0.72 (0.53-0.98) | 0.036 | 0.61 (0.51-0.70) |
| Gender | 0 | Female | 50 (66.7%) |  |  |  |
|  |  | Male | 70 (72.2%) | 1.30 (0.67-2.49) | 0.44 | 0.53 (0.45-0.61) |
| Tegner activity scale preoperative [0-10] | 9 | 1-5 | 26 (59.1%) |  |  |  |
|  |  | 6-10 | 91 (73.4%) | 1.12 (0.97-1.29) | 0.13 | 0.58 (0.49-0.67) |
| ACL; anterior cruciate ligament; OR, Odds ratio; CI, confidence interval; ROC; receiver operating curve.  P-values, OR and Area under ROC-curve are based on original values and not on stratified groups. OR is the ratio for the odds for an increase of the predictor of one unit. *) All tests are performed with univariable logistic regression. | | | | | | |

| **Appedix table A5. Univariable logistic regression model with limb symmetry index ≥ 90% in isokinetic knee flexion strength as dependent outcome.** | | | | | | |
| --- | --- | --- | --- | --- | --- | --- |
| **Predictors** | **n missing** | **Value** | **yes** | **OR (95% CI) LSI Knee Flexion ≥90%** | **p-value** | **Area under ROC-Curve (95%CI)** |
| **Concomitant injuries** | | | | | | |
| Medial meniscus | 0 | Yes | 26 (68.4%) |  |  |  |
|  |  | No | 104 (77.6%) | 1.60 (0.72-3.55) | 0.25 | 0.54 (0.47-0.62) |
| Lateral meniscus | 0 | Yes | 36 (73.5%) |  |  |  |
|  |  | No | 94 (76.4%) | 1.17 (0.55-2.50) | 0.68 | 0.52 (0.44-0.60) |
| Articular cartilage | 0 | Yes | 41 (74.5%) |  |  |  |
|  |  | No | 89 (76.1%) | 1.09 (0.52-2.28) | 0.83 | 0.51 (0.43-0.59) |
| Medial collateral ligament | 0 | Yes | 6 (66.7%) |  |  |  |
|  |  | No | 124 (76.1%) | 1.59 (0.38-6.66) | 0.53 | 0.51 (0.47-0.56) |
| Lateral collateral ligament | 0 | Yes | 1 (100.0%) |  |  |  |
|  |  | No | 129 (75.4%) | 0.00 (0.00-infinity) | 0.99 | 0.50 (0.50-0.51) |
| Meniscus (medial or lateral) | 0 | Yes | 56 (71.8%) |  |  |  |
|  |  | No | 74 (78.7%) | 1.45 (0.72-2.92) | 0.29 | 0.55 (0.46-0.63) |
| **Surgery-related factors** | | | | | | |
| Graft choice | 7 | Hamstring tendon | 109 (73.6%) |  |  |  |
|  |  | Patella tendon | 20 (90.9%) | 3.58 (0.80-16.00) | 0.096 | 0.55 (0.51-0.60) |
| **Anthropometrics** | | | | | | |
| Age at index ACL reconstruction (OR per 10 units) | 0 | 12-<25 | 59 (74.7%) |  |  |  |
|  |  | 25-<35 | 45 (84.9%) |  |  |  |
|  |  | 35-58 | 26 (65.0%) | 0.87 (0.63-1.21) | 0.40 | 0.52 (0.41-0.62) |
| Gender | 0 | Female | 54 (72.0%) |  |  |  |
|  |  | Male | 76 (78.4%) | 1.41 (0.70-2.83) | 0.34 | 0.54 (0.45-0.63) |
| Tegner activity level preoperative [0-10] | 9 | 1-<6 | 27 (61.4%) |  |  |  |
|  |  | 6-10 | 101 (81.5%) | 1.19 (1.02-1.38) | 0.029 | 0.62 (0.52-0.71) |
| ACL; anterior cruciate ligament; OR, Odds ratio; CI, confidence interval; ROC; receiver operating curve.  P-values, OR and Area under ROC-curve are based on original values and not on stratified groups. OR is the ratio for the odds for an increase of the predictor of one unit. *) All tests are performed with univariable logistic regression. | | | | | | |
